# Hemorrhage from the Navel after Detachment of the Funis*Read before the Buffalo Medical Union.

**Published:** 1883-04

**Authors:** U. C. Lynde

**Affiliations:** Buffalo, N. Y.


### Hemorrhage from the Navel after Detachment of the Funis.*

By U. C. Lynde, M. D., Buffalo, N. Y.

The cord is usually detached after the lapse of five or six days from the birth of the child. Hemorrhage from the stump is uncommon; but when it occurs, it is fatal, unless checked by surgical means. We are not now considering those cases in which there is but a mere oozing of blood, but those only from which there is an actual flow of blood. Most practitioners of large obstetrical experience have met with cases that have proven fatal, notwithstanding all their efforts to arrest the hemorrhage. This hemorrhage takes place from the umbilical arteries, and, owing to the soft and yielding parts beneath, com-

* Read before the Buffalo Medical Union.

presses are of no avail. Collodion, astringents, styptics, escharotics, have all been tried in vain. Some of these deceive the practitioner by temporarily checking or arresting the hemorrhage which returns in his absence and proves fatal to his patient. Dr. Gross recommends the twisted suture; but, the conformation and tenderness of the parts, together with the susceptibility of the patient, render this method objectionable. Dr. Francis Minot, of Boston, gives us, as reported by Dr. Gross, a published list of forty-six cases, in which thirty-nine proved fatal. This list probably includes some cases of mere oozing of blood. Dr. Gross mentions four fatal cases in his own practice. He does not tell us how many he has had. Having lost my first patient, after resorting to the usual means for arresting hemorrhage at this site, I resolved that if ever I had another, I would not hesitate to use such means as would ensure the life of my patient as against hemorrhage, at least. My next patient had bled until it was about as white as a sheet when I first saw it. After arming a straight, round needle, having a large eye, with surgeons silk as large as could be drawn through it, my patient was placed upon a pillow, on his back, upon a table, and chloroform administered. A common tenaculum was inserted as close to the stump as practicable, at the apex of the inverted hollow cone, and, by gentle traction, this cone or cup was inverted, its apex being made convex and pointing toward the operator; and, at the same time being made to rise above the level of the surrounding surface of the abdomen. The needle was now made to transfix the stump from which the funis had been detached, care being taken not to dip deep enough to wound the peritoneum. The double ligature was cut, leaving both ends long enough to tie conveniently. Again the stump was transfixed; this time at right angles to the first, and the threads cut off as before. The threads were carefully separated and tied so as to include the entire stump, but tied only tight enough to stop the hemorrhage, and *not* tight enough to strangulate the parts that were included. The ends of the ligatures were now cut close to the knots, the tenaculum removed, and the parts allowed to resume their normal position, that of a hollow cone with the apex pointing toward the abdominal viscera. After a few days the thread was cut and drawn out, no hemorrhage followed and my little patient survived and is now almost a man. Since then I have operated on two cases with like results. In making this little operation the *size* of the thread and the *kind of needle* used are important. The *inversion* of the *hollow cone* and the *transfixion* of the *stump* are to be carefully performed. Tying the ligatures is a matter more of observation than of sensation, as the entire cessation of the hemorrhage is the surgeon's guide, unless holding the parts with the tenaculum has so checked the bleeding as to destroy this means of judging, and then the surgeon must judge as best he can of the force he uses by the sensation upon his fingers.

